# Large-Scale Patterns of Turnover and Basal Area Change in Andean Forests

**DOI:** 10.1371/journal.pone.0126594

**Published:** 2015-05-14

**Authors:** Selene Báez, Agustina Malizia, Julieta Carilla, Cecilia Blundo, Manuel Aguilar, Nikolay Aguirre, Zhofre Aquirre, Esteban Álvarez, Francisco Cuesta, Álvaro Duque, William Farfán-Ríos, Karina García-Cabrera, Ricardo Grau, Jürgen Homeier, Reynaldo Linares-Palomino, Lucio R. Malizia, Omar Melo Cruz, Oriana Osinaga, Oliver L. Phillips, Carlos Reynel, Miles R. Silman, Kenneth J. Feeley

**Affiliations:** 1 Consorcio para el Desarrollo Sostenible de la Ecoregión Andina (CONDESAN), Quito, Ecuador; 2 Universidad Técnica Particular de Loja, Loja, Ecuador; 3 Consejo Nacional de Ciencias de Investigaciones Científicas y Técnicas (CONICET), Buenos Aires, Argentina; 4 Instituto de Ecología Regional (IER), Universidad Nacional de Tucumán, Tucumán, Argentina; 5 Facultad de Ciencias Forestales, Universidad Nacional Agraria La Molina, Lima, Perú; 6 Universidad Nacional de Loja, Loja, Ecuador; 7 Laboratorio de Servicios Ecosistémicos y Cambio Climático, Jardín Botánico de Medellín, Medellín, Colombia; 8 Departamento de Ciencias Forestales, Universidad Nacional de Colombia, Medellín, Colombia; 9 Department of Biology, Wake Forest University, Winston-Salem, North Carolina, United States of America; 10 Plant Ecology, University of Göttingen, Göttingen, Germany; 11 Facultad de Ciencias Agrarias, Universidad Nacional de Jujuy, Jujuy, Argentina; 12 Fundación ProYungas, Jujuy, Argentina; 13 Grupo de investigación en Biodiversidad y Dinámica de Ecosistemas Tropicales, Universidad del Tolima, Bogotá, Colombia; 14 School of Geography, University of Leeds, Leeds, United Kingdom; 15 International Center for Tropical Botany, Department of Biological Sciences, Florida International University, Miami, Florida, United States of America; Berkeley, UNITED STATES

## Abstract

General patterns of forest dynamics and productivity in the Andes Mountains are poorly characterized. Here we present the first large-scale study of Andean forest dynamics using a set of 63 permanent forest plots assembled over the past two decades. In the North-Central Andes tree turnover (mortality and recruitment) and tree growth declined with increasing elevation and decreasing temperature. In addition, basal area increased in Lower Montane Moist Forests but did not change in Higher Montane Humid Forests. However, at higher elevations the lack of net basal area change and excess of mortality over recruitment suggests negative environmental impacts. In North-Western Argentina, forest dynamics appear to be influenced by land use history in addition to environmental variation. Taken together, our results indicate that combinations of abiotic and biotic factors that vary across elevation gradients are important determinants of tree turnover and productivity in the Andes. More extensive and longer-term monitoring and analyses of forest dynamics in permanent plots will be necessary to understand how demographic processes and woody biomass are responding to changing environmental conditions along elevation gradients through this century.

## Introduction

Our understanding of the responses of tropical forests to environmental factors is still limited [1–3]. Indeed, general patterns of forest dynamics and productivity in the Andes Mountains in particular remain poorly characterized due to the scarcity of studies, as well as the complexity of environmental variation in these topographically complex systems [4–6]. This lack of knowledge is troubling since the Andes are among the most important areas for biological conservation in the world [7] and deliver valuable environmental services (e.g., water provision and carbon storage) to large human populations [8, 9]. In addition, the ecological functioning of the Andes is tightly linked to that of the Amazonian rainforests lying to their eastern flank, which is another area critical for biological conservation. The lack of knowledge about the influence of climate on dynamics and productivity of tropical forests is of special concern in the face of on-going and future climate change [10].

Tree turnover and primary productivity are controlled to a large extent by environmental factors. In mountain ecosystems, tree turnover (the mortality and recruitment of stems) and net primary productivity (gross production of fixed carbon) both tend to decrease with elevation and lower environmental temperatures [11–15]. The influence of precipitation on tree turnover and productivity is less clear than that of temperature. In general, forest productivity increases with precipitation but the relationship is not linear [16], and high precipitation may have negative effects on plant production in some tropical mountain forests [17]. There is increasing evidence that temperature and precipitation patterns are shifting in the Andean region [18–20], and that Andean forests are already responding to environmental changes. For example, trees in the Peruvian Andes have shifted their distributions upslope over the past decades potentially in response to rising temperatures [21].

The main objective of this paper is to describe how rates of tree turnover, tree growth and basal area net change, a simple proxy for woody productivity, are affected by environmental and geographic variation in the Andes. Thus far, studies on this subject have only been conducted over relatively small geographic scales [12, 13, 15, 22, 23]. Here we used field data collected from permanent forest monitoring plots distributed widely from Colombia to northern Argentina. Using data from these plots, we investigated how tree turnover and growth rates vary across gradients of latitude, elevation, temperature, and precipitation within two regions: the North-Central Andes and North-Western Argentina. Moreover, we evaluated changes of basal area as a function of environmental variation in 32 permanent plots in the Northern-Central Andes. This is the first study to characterize and synthesize patterns of forest dynamics across a large spatial scale in Andean forests.

## Materials and Methods

**Fig 1:**
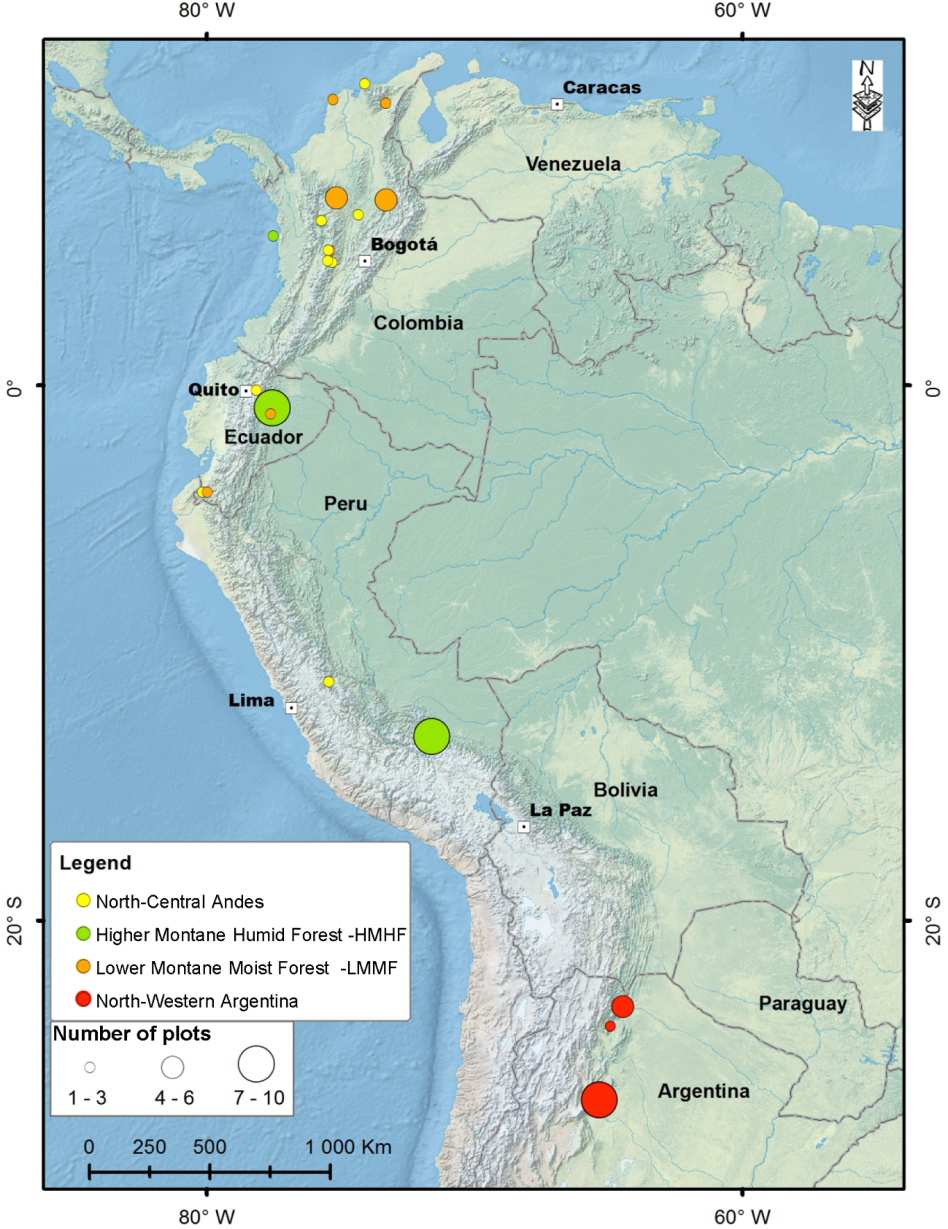
Distribution of the 63 permanent forest plots used in this study. North-central Andes plots were only used for analyses of tree turnover.

This study was conducted using data collected from 63 permanent forest monitoring plots located across a latitudinal gradient spanning approximately 4000 km (Fig 1) in Colombia, Ecuador, Peru and Argentina. The plots were distributed across a large range of elevations, from 57 to 3940 m above sea level (Fig 1 and S1 Table). Fifty-two study plots were located in the Andean cordillera above 500 m (1812 m ± 121), while 11 were in the Amazonian and Caribbean lowlands adjacent to the cordillera.

The permanent plots had a uniform distribution across the elevation gradient. The mean elevation of the North-Central Andean plots was 1801 m (range = 3883 m, median 1980 m). Only the permanent plots in southern Peru were placed along a large elevation gradient, from 1800 to 3450 m above sea level (Fig 1). The other sites with a high concentration of permanent plots did not have strong altitudinal variation. On average, Argentinian plots were located at 886 m above sea level (range = 1285, median = 898).

The plots varied in size from 0.4 to 1 ha (S1 Table) and were established by independent teams of researchers for different purposes; however, all plots share a common core set of installation and census methods stated in or comparable to the RAINFOR protocol [24]. Plots were established in mature forests with no, or only minor, signs of recent human influence, and also avoiding natural landscape-scale disturbance processes such as landslides. In each of the study plots, all trees ≥ 10 cm of diameter at breast height (DBH) were identified and measured for diameter at least twice. First censuses were conducted between 1996 and 2009 (mean year of first census = 2003.1±0.34), and plot re-censuses took place between 2000 and 2012 (mean year of last census = 2008.3 ±0.31). On average, plots were thus resampled 5.2 years after their establishment (minimum and maximum resampling periods = 1.75 and 10.16 years, respectively). We used DBH measurements to estimate the total basal area of individuals (cross sectional area of stems at breast height; m^2^ ha^-1^) in each census.

There was a negative relationship between altitude and temperature in our data set corresponding to lapse rates of 4.9°C and 5.1°C for every 1 km increase in elevation in the North-Central Andes (y = 27.44–0.0049 * elevation (m), *P*<0.001) and in the Argentinean sites (y = 23.8–0.0051 * elevation (m), *P*<0.001). Only in the North-Central Andes precipitation rates were somewhat negatively related with elevation (y = 2971.3–0.2 * elevation (m), *P*>0.05), as in the Argentinian sites there was no relationship between these two variables (y = 1109–0.01 * elevation (m), P>0.05).

We calculated demographic rates for each plot using the following equations: Annual mortality rates were estimated as m = 1-[1- (N_0_—N_1_)/ N_0_]^1/t^ * 100, annual recruitment rates (trees reaching 10 cm DBH): r = 1-(1- N_r_—N_1_)^1/t^ * 100; where N_0_ is the number of individuals alive in the initial census, N_1_ is the number of individuals surviving to the second census, N_r_ is the number of individuals recruited between censuses, t corresponds to time in years. Turnover was estimated as the mean of tree mortality and recruitment [25]. Basal area net change was estimated as BAn = ((BA_1_-BA_0_)/ BA_0_)/t * 100 and relative tree growth rate was calculated as g = ((BA_1_-BA_r_)-(BA_0_-BA_d_) / (BA_0_- BA_r_))/t * 100 BA_0_ and BA_1_ correspond to basal area values at the initial and last census, respectively; BA_r_ is the basal area of trees recruited, and BA_d,_ is the basal area of dead trees in the last census, t is time in years [26]. Tree growth was estimated as changes of the basal area of trees surviving through the census period. To correct for census interval effects in estimates of turnover for plots in the North-Central Andes we used the formula *ʎ*_*corr*_ *= ʎ t* ^*0*.*08*^, where *ʎ*_*corr*_ is turnover rate standardized to a 1-year census interval, *ʎ* is raw turnover rate, ant *t* is the length of census interval in years [27]. Demographic rates for each of the individual study plots are presented in the S2 and S3 Tables.

We analysed the relationships between our measures of forest demography and several environmental and geographic variables (Table 1). Our analyses included the following information for each plot: latitude, elevation, total annual precipitation, minimum temperature (i.e., average monthly minimum temperatures), and a pluvio-thermic index (Iod2). Iod2 = Pp/Tp where Pp is the total precipitation in mm, and Tp is the sum of the monthly temperature higher than 0°C in tenths of °C for the two consecutive driest months of the year [28]. Pluvio-thermic (or ombrothermic) indices express the relationship between precipitation and temperature at a given site and thus are used to classify vegetation in categories that range from dry to hyper-humid (dry sites have low scores and humid sites have high scores), providing a way to express the outcome in one index of the large variations in rates of both moisture supply and moisture loss that exist in the geographically highly variable climates of our study region. Latitudinal location, minimum annual temperatures, and the pluvio-thermic index were included in the statistical models described below to account for extremes of environmental variation, which were partly related to the geographic distribution of our forest plots. Minimum annual temperature, total annual precipitation, and the variables needed to calculate the pluvio-thermic index were obtained from the WorldClim extrapolated climate database (www.worldclim.org) [29]. For some plots, total annual precipitation rates were recorded at nearby meteorological stations. Data analyses were conducted using the statistical package JMP [30]. The ethics statement is presented in the S1 File.

**Table 1:** Environmental and demographic forest variables in the North-Central Andes, and North-Western Argentina.

Parameter	North-Central Andes Mean ± SE (Min-Max) n = 45	North-Western Argentina Mean ± SE (Min-Max) n = 18
**Latitude**	0.74 + 1.17	-25.15 + 0.44
	(11.26 –-13.11)	(-22.27 –-26.76)
**Total annual precipitation (mm)**	2620 + 169	1084 + 12
	(1007–5977)	(925–1150)
**Minimum annual temperature (°C)**	12.22 + 0.85	6.43 + 0.28
	(0.9–22.6)	(4.2–8.5)
**Pluvio-thermic index (Iod2)**	5.43 + 0.64	0.60 + 0.05
	(0.05–16.76)	(0.32–1.01)
**Tree turnover rate (% yr^-1^)**	1.88 + 0.11	2.42 + 0.24
	(0.25–3.21)	(1.18–5.54)
**Tree growth (m^2^ ha^-1^ yr^-1^)**	0.41 + 0.03	0.41 + 0.03
	(0.12–0.92)	(0.27–0.71)
**Relative tree growth (% ha^-1^ yr^-1^) ^a^**	1.86 + 0.15	0.33 + 0.04
	(0.45–4.16)	(0.14–0.74)
**Basal area net change (% ha^-1^ yr^-1^) ^a^**	0.31 + 0.21	0.55 + 0.23
	(-2.44–2.86)	(-1.09–3.48)

^a^ n = 32 North-Central Andean plots

### Data Analyses

We used subsets of our data base to explore aspects of forest demography in areas with different environmental or geographical features. Hence, responses of tree turnover, growth, and basal area net change to environmental variation were examined separately for the 45 North-Central Andean plots and for the 18 North-Western Argentina plots. In addition, patterns of forest demography were examined for 32 North-Central Andean plots, considering two forest groups, as explained below.

We conducted Principal Components Analysis (PCA) to characterize the environmental heterogeneity recorded in our 63 forest monitoring plots in fewer and uncorrelated variables. The PCA included data on elevation, latitude, minimum temperature, total annual precipitation, and the pluvio-thermic index (Table 2). We conducted linear regression analyses to examine the relationships between the first two PCA axis and tree turnover, tree growth rates, and basal area net change. Analyses were conducted separately for two groups of plots, those located in the North-Central Andes (Colombia to Peru), and plots in North-Western Argentina.

**Table 2:** Loadings and variation explained in two Principal Components Analysis using five environmental variables recorded in 63 permanent plots, and for a subset of 32 permanent plots located in the North-Central Andes.

	All plots (n = 63)		North-Central Andean plots (n = 32)	
	Axis 1	Axis 2	Axis 1	Axis 2
**Latitude**	0.83683	-0.07509	0.76207	-0.34325
**Elevation**	0.10485	0.92060	-0.94232	-0.15800
**Total annual precipitation**	0.76785	0.14083	-0.01932	0.94545
**Pluvio-thermic index (Iod2)**	0.75961	0.44616	0.09185	0.74545
**Minimum annual temperature**	0.64159	-0.74929	0.96992	0.06443
**Variation explained**	45%	33%	48%	32%

**Fig 2:**
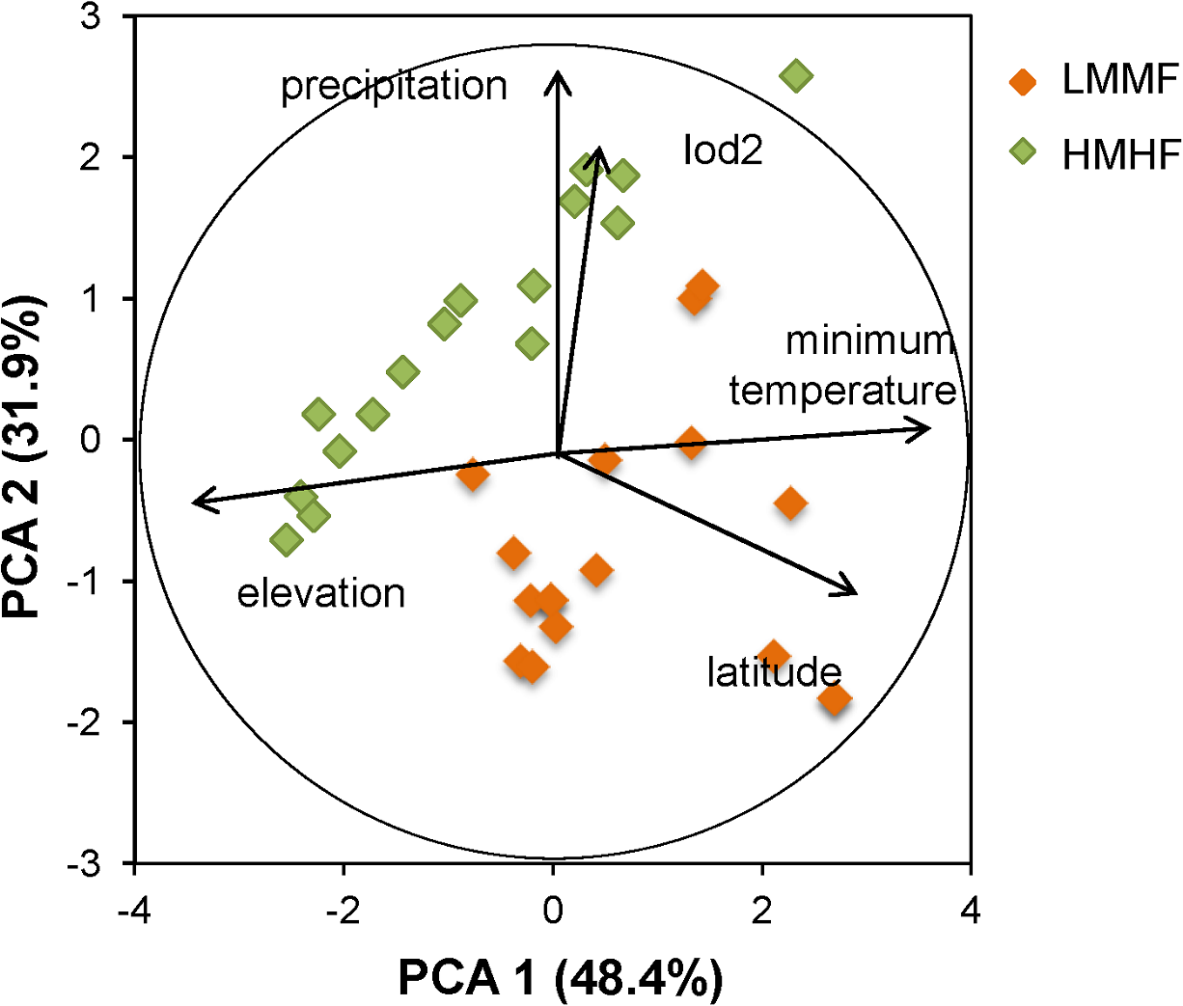
Classification of 32 North-Central Andean permanent plots. Classification according to the plot’s scores in the first two factors of a Principal Component Analysis.

A second PCA included the same environmental variables considered above, but only for 32 northern plots (Fig 1). The scores of each plot in the first two PCA axes were used to identify two groups of plots with contrasting environmental conditions (Figs 1 and 2, Tables 2 and 3). The group of Higher Montane Humid Forests (HMHF) included permanent plots located at higher elevation, with lower minimum temperature, receiving high precipitation rates, and higher pluvio-thermic scores compared to Lower Montane Moist Forests (LMMF). Latitude also differed between forest groups; HMHFs were located toward the south (lower latitude) and LMMFs occurred at northern locations (higher latitude). Each forest group had 16 permanent plots. We used ANOVA to compare environmental, geographic features, and forest demography between both groups (Table 3).

**Table 3:** Descriptive statistics of the geographic, environmental, and demographic variables of Higher Montane Humid Forests (HMHF) and Lower Montane Moist Forests (LMMF).

	LMMF Mean ± SE (Min-Max) n = 16	HMHF Mean ± SE (Min-Max) n = 16	*F*_*1*, *31*_	*R* ^*2*^	*P*
**Elevation (m)**	1453.6 ± 263.3	2146.2 ± 247.1	3.7	0.10	0.064
	(57–2970)	(60–3940)			
**Minimum annual temperature (°C)**	14.36 ± 1.35	9.57 ± 1.26	6.6	0.18	**0.015**
	(7–22.6)	(0.9–21.1)			
**Latitude**	5.75 ± 1.05	-6.47 ± 1.74	35.8	0.54	**<0.001**
	(-3.99–10.67)	(-13.11–5.58)			
**Total annual precipitation (mm)**	2095.5 ± 227.1	3630.4 ± 260.4	19.7	0.39	**<0.001**
	(1007–3677)	(1403–5977)			
**Pluvio-thermic index (Iod2)**	4.15 ± 0.6	7.07 ± 1.3	4.3	0.11	0.067
	(0.6–8.6)	(0.6–16.7)			
**Basal area change (% ha^-1^ yr^-1^)**	0.84 ± 0.26	-0.19 ± 0.25	7.9	0.22	**0.008**
	(-0.71–2.86)	(-2.44–1.11)			
**Tree growth (m^2^ ha^-1^ yr^-1^)**	0.39 ± 0.02	0.36 ± 0.03	0.5	0.01	0.489
	(0.23–0.56)	(0.12–0.75)			
**Relative tree growth (% ha^-1^ yr^-1^)**	2.23 ± 0.18	1.32 ± 0.11	18.0	0.38	**0.001**
	(1.48–4.16)	(0.45–2.28)			
**Tree recruitment rate (% yr^-1^)**	1.71 ± 0.23	0.99 ± 0.13	6.8	0.18	**0.013**
	(0.47–3.45)	(0.00–2.10)			
**Tree mortality rate (% yr^-1^)**	2.27 ± 0.29	2.24 ± 0.30	0.002	0.01	0.959
	(0.77–5.39)	(0.52–4.74)			

Statistically significant results of ANOVAs comparing the two forest groups are presented in bold.

## Results

The PCA, including 63 permanent plots, produced a first PCA axis (PCA 1) that captured 45% of the environmental variation and was positively correlated with latitude, total annual precipitation, and high pluvio-thermic scores. The second PCA axis (PCA 2) described 33% of the environmental variation and was positively correlated with elevation and negatively correlated with minimum annual temperature. Combined, both axes explained 78% of the environmental variation (Table 2).

### North-Central Andes

**Fig 3:**
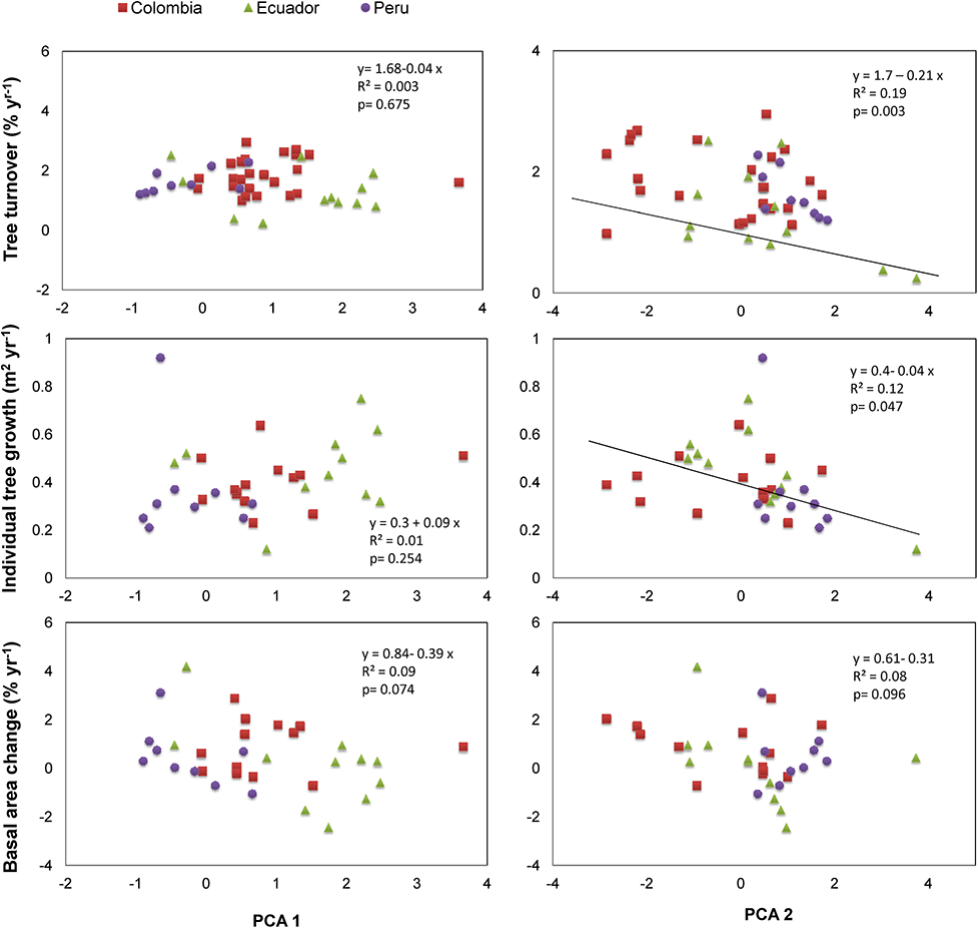
Linear regressions of forest demographic variables as a function of the first two factors of a Principal Component Analysis.

Our results showed that forest demography in this region was affected by the environmental variation captured in PCA 2. Rates of tree turnover, total and relative growth, and basal area net change were negatively related to PCA 2, indicating that these demographic variables decreased with increasing elevation and declining minimum temperatures (Fig 3, Table 4, S4 Table). None of our demographic variables were related to PCA 1.

**Table 4:** Summary of the results of Linear Regression analyses using PCA axes as predictive variables of forest demography for plots in North-Central, and North-Western Argentina.

		PCA 1			PCA 2		
	Variables	*R* ^*2*^	Estimate	*P*	*R* ^*2*^	Estimate	*P*
**North-Central Andes**	Tree turnover (% yr^-1^)	0.003	-0.04	ns	0.19	-0.21	0.003
	Tree growth (m^2^ yr^-1^)	0.01	0.09	ns	0.12	-0.04	0.047
	Relative tree growth (% yr^-1^)	0.01	0.05	ns	0.32	-0.38	<0.001
	Basal area change (% yr^-1^)	0.09	0.39	ns	0.08	-0.31	ns
**North-Western Argentina**	Tree turnover (% yr^-1^)	0.08	-0.05	ns	0.01	-0.24	ns
	Tree growth (m^2^ yr^-1^)	0.13	-0.34	ns	0.29	1.68	0.021
	Relative tree growth (% yr^-1^)	0.04	0.15	ns	0.05	0.25	ns
	Basal area change (% yr^-1^)	0.22	-0.06	0.03	0.13	0.15	ns

### North-Western Argentina

**Fig 4:**
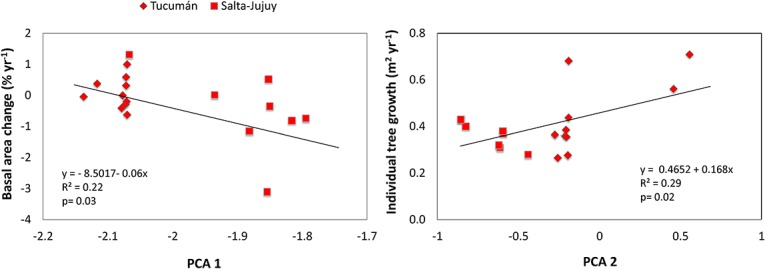
Linear regressions of forest demographic variables in North-Western Argentina. Basal area net change and individual tree growth and as a function of the first two factors of a Principal Component Analysis.

We found a negative relationship between PCA 1 and basal area net change, as it decreased with increasing latitude, annual precipitation and pluvio-thermic scores (Fig 4, Table 4, S4 Table). We also found that in this region tree growth was positively related to PCA 2, thus it increased with elevation and with minimum environmental temperatures. Neither tree turnover nor relative growth was related to our PCA axes (S1 Fig).

### North-Central Andes: Higher Montane Humid and Lower Montane Moist Forests

The first PCA axis captured 48% of the environmental variation, and it was positively correlated to low elevation, high minimum temperatures, and high latitude (Table 2, Fig 2). The second PCA axis explained 32% of the variation and was positively correlated with high annual precipitation rates and high pluvio-thermic scores. Therefore, the first two axes captured 80% of the environmental variation.

Our analyses showed that LMMFs increased in basal area over the study period (mean = 0.84 ± 0.26%), whereas HMHFs did not have statistically significant changes in net basal area change (mean = -0.19 ± 0.25%), and that these differences were significantly different (Table 3). In addition, LMMFs also had significantly higher relative tree growth rates than HMHFs, although this pattern did not hold for total tree growth rates (Table 3). In addition, tree recruitment was faster in LMMFs than at HMHFs. Mortality rates did not differ between forest groups (Table 3). Within forest groups, tree mortality was significantly higher than tree recruitment in HMHFs (*F*_1,15_ = 3.76, *P*<0.001), but these demographic rates did not differ in LMMFs (*F*_1,15_ = 1.13, *P* = 0.275).

## Discussion

This study shows that the rates of tree turnover and basal area net change of Andean forests are strongly related to large-scale variation in geographic and environmental factors. Moreover, we found contrasting patterns of basal area change in forests with different environmental features. Tree turnover and tree growth decreased with elevation and with lower minimum temperatures in the North-Central Andean forests (Fig 3). The same tendency was observed for basal area net change. Thus, woody growth declined with elevation at this latitudinal zone. This pattern has been found several times in temperate regions [11, 31], and at smaller spatial scales in the Andes [12, 13, 15, 23, 32]; however, this is the first time this elevational trend is documented across a large spatial scale in the tropical Andes.

Given the strong connection between elevation and temperature, the decrease in tree growth at high elevations is possibly due to effects of low temperature on plant metabolism [33] and its indirect effect on nutrient cycling [23, 32]. Other abiotic factors that reduce plant growth often vary with elevation and may contribute to explain this trend. For example, deceasing soil fertility with elevation reduces productivity at higher altitudes in certain Andean forests [23, 32], and at other mountain areas [31]. In the same way, increasing cloudiness at higher elevations decreases direct solar radiation and reduces productivity [14, 34].

Biotic factors can also increase tree turnover at lower elevations. For example high rates of tree mortality at low elevations may be due to increased aboveground competition among tree species, stronger top-down regulation exerted by herbivores, disease [35], and parasitism [31]. In addition, tree communities at lower elevations generally have higher relative abundances of tree species that favour growth over defence strategies, which results in high recruitment rates [35].

It is interesting to note that tree turnover, tree growth rates, and basal area net change were not associated with PCA 1, which described a gradient of low to high precipitation, pluvio-thermicity, and latitude (Fig 2). The lack of demographic responses to these environmental factors is likely related to methodological issues, rather than a lack of their effect on tree turnover and basal area net change, as it has been demonstrated in previous studies [17, 36, 37]. Our dataset is certainly an incomplete sample of the precipitation gradient in the Andes. In addition, we used modeled rather than field climatic variables, which may be problematic due to the complexity of modeling climatic variables in mountain regions [38], where ‘horizontal precipitation’ and cloud cover may affect the amount of water available for plant growth [34]. Using long-term data taken within narrower latitudinal zones in the Andes may be a first step to understand how precipitation affects forest demography in this region.

The patterns of forest demography in North-Western Argentina differed from those of the North-Central Andes. Here basal area net change decreased with higher precipitation, pluvio-thermicity, and latitude (PCA 1) and tree growth increased with elevation and with lower minimum temperatures (PCA 2) (Table 2, Fig 3). These unexpected patterns are explained by the abiotic factors considered and ecological processes related to forest recovery. The results were driven to a large extent by the more humid Tucuman plots located at higher elevations (1000–1700 m), and at lower latitude (Fig 4). Tucuman plots had higher pluvio-thermic index scores (but not higher precipitation rates) compared to the other two Argentinian sites, Salta and Jujuy. These plots had a 50% increase in stem density and 6% in basal area between 1992 and 2007, which has been attributed to forest recovery after the removal of livestock (i.e., cattle and horses) [39] once the area was declared a natural reserve in 1973 [40]. Hence, higher tree growth rates at higher elevation sites may also be due to the recovery of populations of trees palatable to herbivores, which may favor growth over persistence trade-offs.

Taken together, our results indicate that combinations of abiotic and biotic factors that vary across elevation gradients are important determinants of tree turnover and productivity in the Andes. It is noteworthy that this pattern emerges despite the large variation in species composition (among many other variables) associated to the geographical variation considered in the study. It is likely that the effect of our environmental variables on forest demography vary in strength and even in sign at narrower latitudinal zones, that are represented as different countries or sites in this study (Figs 3 and 4). In addition, in the North-Western Argentina, where we considered a narrower latitudinal zone, these environmental controls of tree turnover and productivity were modified by past land-use and ecological recovery.

### North-Central Andes: Higher Montane Humid and Lower Montane Moist Forests

Our results overall indicate a balance between stem recruitment and mortality at lower elevation moist forests (LMMFs), while all have increased basal area. In contrast, at higher elevations the plots showed an excess of mortality over tree recruitment, and no significant change in basal area (Table 3). The increase in basal area at lower elevations could be caused by a number of reasons. It is for example consistent with evidence that Amazonian and other lowland forests have experienced net biomass increases in recent decades [1, 41–43], which has been attributed to increased nutrient deposition, atmospheric CO_2_, and shifts on precipitation regimes [44], but other factors may also be in play in the Andes. The lack of basal area net change at higher elevations and excess of mortality over recruitment suggests negative environmental impacts.

Globally, as well as in tropical forests, drought and higher environmental temperatures affect tree mortality [45, 46], and reduce aboveground productivity [47]. Between 2000–2012, a period of sampling of most of our permanent plots, some areas in the Northern and Central Andes experienced lower precipitation rates than those recorded in previous decades [47]. Thus, higher environmental temperatures could be interacting with lower precipitation rates to affect tree performance and mortality in HMHFs. Lower precipitation rates or short drought periods can enhance tree mortality even in forests that are not considered to be water-limited [48–50]. Hydraulic stress can kill trees through xylem embolism or carbon starvation and by enhancing negative effects of insect attacks and disease [46].

High tree mortality rates reflect various biological responses to environmental change in our study area. For instance, upward migration of Andean trees, possibly linked to higher environmental temperatures [21] may not be a process in which mortality and recruitment rates occur at the same time. Periods of high mortality and low recruitment of species sensitive to higher environmental temperatures may take place. During such periods, low recruitment rates may be magnified by lagged migration of species adapted to higher environmental temperatures. More detailed, longer-term analyses of forest dynamics in permanent plots are clearly necessary to understand how demographic processes and woody biomass are responding to changing environmental conditions along elevation gradients.

Taken together our results suggest a high sensitivity of Andean forest dynamics to climate. Perhaps more importantly, these results suggest that Andean forests are responding to the changes in climate that are already occurring in this region and that are predicted to occur in the future. Further, Andean montane forests seem to be responding to their changing environment in different ways than lowland tropical forests. In the Andes, mean annual temperatures increased 0.34°C decade^-1^ over the period from 1974–1998 [51], a rate much higher than that observed for the Neotropical region as a whole [52]. Moreover, temperature increases have been more pronounced at higher elevations in the Andes [53], which may cause stronger biological responses in these areas. These findings provide a baseline for future studies exploring the responses of Andean tree species to environmental variation and to future climate scenarios. This on-the-ground monitoring network is still at an early stage. Detailed analyses of the existing information in the context of forest responses to directional climate change, and further field monitoring will help to understand if the small changes observed so far are part of a larger, cyclical pattern, perhaps induced by decadal climate fluctuations.

## Supporting Information

S1 Table Location, geographical and environmental features, and establishment information of the permanent plots used in this study. Country codes: Argentina (ARG), Colombia (COL), Ecuador (ECU), Peru (PER). Authors codes: AD: A. Duque, AM: A. Malizia, CB: C. Blundo, EA: E. Álvarez, JC: J. Carilla, JH: J. Homeier, KJF: K.J. Feeley, LM: L. Malizia, NA: N. Aguirre, OO: O. Osinaga, SB: S. Báez, ZA: Z. Aquirre, WF: W. Farfán, RLP: R. Linares-Palomino.
(DOCX)

S2 Table Demographic rates, and dominant or most common species of the 45 permanent plots located in the North-Central Andes. “Forest group” indicates the classification of the forest plot in one of the groups: HMHFs = Higher Montane Humid Forest, LMMFs = Lower Montane Moist Forests. Country codes: COL = Colomobia, ECU = Ecuador, PER = Peru. Upperscripts on the Plot code indicate the methodological protocol used. The scientific nomenclature was updated according to the Global Biodiversity Information Facility databases (GBIF; www.gbif.org).
(DOCX)

S3 Table Demographic rates, and dominant or most common species of the 18 permanent plots located in South-Western Argentina. Scientific nomenclature after the Global Biodiversity Information Facility databases (GBIF; www.gbif.org).
(DOCX)

S4 Table Results of the Linear regression analyses using PCA factors as predictors of forest demography in North-Central Andean, and North-Western Argentina. (DOCX)

S1 File Ethics statement. (DOCX)

S1 Fig Graphs of the Linear regression analyses using PCA factors as predictors of forest demography in North-Western Argentina. (TIFF)
