# Supplementary material for: Large-Scale Patterns of Turnover and Basal Area Change in Andean Forests
Source: PLoS One. 2015 May 14;10(5):e0126594. doi: 10.1371/journal.pone.0126594 (PMC4431807; doi:10.1371/journal.pone.0126594)
Supplement: S1 Table — Country codes: Argentina (ARG), Colombia (COL), Ecuador (ECU), Peru (PER). Authors codes: AD: A. Duque, AM: A. Malizia, CB: C. Blundo, EA: E. Álvarez, JC: J. Carilla, JH: J. Homeier, KJF: K.J. Feeley, LM: L. Malizia, NA: N. Aguirre, OO: O. Osinaga, SB: S. Báez, ZA: Z. Aquirre, WF: W. Farfán, RLP: R. Linares-Palomino. (DOCX) [file pone.0126594.s001.docx]

**S1 Table. Location, geographical and environmental features, and establishment information of the permanent plots used in this study.** Country codes: Argentina (ARG), Colombia (COL), Ecuador (ECU), Peru (PER). Authors codes: AD: A. Duque, AM: A. Malizia, CB: C. Blundo, EA: E. Álvarez, JC: J. Carilla, JH: J. Homeier, KJF: K.J. Feeley, LM: L. Malizia, NA: N. Aguirre, OO: O. Osinaga, SB: S. Báez, ZA: Z. Aquirre, WF: W. Farfán, RLP: R. Linares-Palomino.

| Plot | Country | Code plot | Latitude | Longitude | Elevation (m) | Precipitation (mm yr-1) | Pluvio-seasonal Index (Iod2) | Minimum temperature ( C) | Year of establishment | Census interval (yrs) | Paper author |
| --- | --- | --- | --- | --- | --- | --- | --- | --- | --- | --- | --- |
| 1 | Argentina | AbraGrande550 | -23.12134 | -64.47196 | 465 | 1056 | 0.4 | 8.1 | 2003 | 5 | CB, LM |
| 2 | Argentina | Ha1 | -26.76283 | -65.33323 | 973 | 1090 | 0.75 | 6 | 2002 | 5 | AM, JC,OO |
| 3 | Argentina | Ha2 | -26.76202 | -65.33314 | 985 | 1090 | 0.75 | 6 | 2002 | 5 | AM, JC,OO |
| 4 | Argentina | Ha3 | -26.76114 | -65.3332 | 980 | 1090 | 0.75 | 6 | 2002 | 5 | AM, JC,OO |
| 5 | Argentina | Ha4 | -26.76283 | -65.3325 | 996 | 1090 | 0.75 | 6 | 2002 | 5 | AM, JC,OO |
| 6 | Argentina | Ha5 | -26.76194 | -65.33252 | 1002 | 1090 | 0.75 | 6 | 2002 | 5 | AM, JC,OO |
| 7 | Argentina | Ha6 | -26.76098 | -65.33255 | 1001 | 1090 | 0.75 | 6 | 2002 | 5 | AM, JC,OO |
| 8 | Argentina | Km25 | -22.27392 | -63.83928 | 845 | 1088 | 0.38 | 6.8 | 2005 | 4 | AM, JC,OO |
| 9 | Argentina | Mi1 | -26.70049 | -65.33583 | 1750 | 1090 | 0.89 | 4.2 | 2001 | 6 | AM, JC,OO |
| 10 | Argentina | Mi2 | -26.71375 | -65.3372 | 1630 | 1090 | 1.01 | 4.4 | 2001 | 6 | AM, JC,OO |
| 11 | Argentina | norte | -26.76466 | -65.33021 | 910 | 1090 | 0.75 | 6 | 2001 | 5 | AM, JC,OO |
| 12 | Argentina | RíoSecoII | -22.54880 | -63.92555 | 711 | 925 | 0.34 | 7.6 | 2004 | 5 | CB, LM |
| 13 | Argentina | SanMartin600 | -23.7696 | -64.8044 | 595 | 1043 | 0.32 | 7 | 2002 | 6 | CB, LM |
| 14 | Argentina | Sur | -26.76455 | -65.33016 | 886 | 1090 | 0.75 | 6 | 2001 | 5 | CB, LM |
| 15 | Argentina | Tabacal500 | -23.22202 | -64.46175 | 521 | 1150 | 0.37 | 8.2 | 2003 | 5 | CB, LM |
| 16 | Argentina | Tecpetrol500 | -22.93241 | -64.4508 | 524 | 1150 | 0.36 | 8.5 | 2003 | 5 | CB, LM |
| 17 | Argentina | ValleMorado650 | -23.44233 | -64.52046 | 677 | 1150 | 0.35 | 7.1 | 2003 | 5 | CB, LM |
| 18 | Argentina | Yuchan500 | -23.94066 | -64.9052 | 500 | 1043 | 0.34 | 5.9 | 2002 | 6 | CB, LM |
| 19 | Colombia | AMARGAL2 | 5.58 | -77.499 | 60 | 5977 | 10.95 | 21.1 | 2006.41 | 4.55 | AD |
| 20 | Colombia | Angelopolis | 6.15889 | -75.71083 | 2133 | 2047 | 3.84 | 14.3 | 2007 | 2 | EA |
| 21 | Colombia | Besotes | 10.527 | -73.295 | 344 | 1496 | 0.65 | 18.7 | 2006.5 | 4.06 | EA |
| 22 | Colombia | BETULIA1 | 6.918 | -73.301 | 2274 | 1705 | 4.33 | 10.5 | 2001.8 | 8.89 | EA |
| 23 | Colombia | BETULIA2 | 6.918 | -73.301 | 2274 | 1705 | 4.33 | 10.5 | 2001.8 | 8.89 | EA |
| 24 | Colombia | CIMITARRA | 6.375 | -74.321 | 125 | 2526 | 2.81 | 22.1 | 2001.67 | 2.73 | EA |
| 25 | Colombia | COMBEIMA | 4.586 | -75.319 | 2326 | 1860 | 7.04 | 8.9 | 1999 | 6.9 | EA |
| 26 | Colombia | ELCEIBAL | 10.676 | -75.267 | 57 | 1012 | 0.06 | 22.6 | 2001.67 | 6 | EA |
| 27 | Colombia | ElCeibal2 | 10.676 | -75.264 | 57 | 1007 | 0.05 | 22.6 | 2008.04 | 1.75 | EA |
| 28 | Colombia | ELDIVISO | 7.112 | -73.03 | 2281 | 1243 | 2.88 | 8.9 | 2001 | 10.16 | EA |
| 29 | Colombia | ELRASGON | 7.049 | -73.006 | 2331 | 1305 | 3.01 | 8.1 | 2001.02 | 9.71 | EA |
| 30 | Colombia | FARALLONESE | 5.748 | -76.019 | 1914 | 2775 | 6.93 | 13.4 | 1996 | 4.5 | EA |
| 31 | Colombia | FARALLONESU | 5.748 | -76.019 | 1914 | 2775 | 6.93 | 13.4 | 1996 | 4.5 | EA |
| 32 | Colombia | Laforzosa-A | 6.99306 | -75.14556 | 1750 | 3175 | 4.78 | 12.6 | 2006 | 3 | AD |
| 33 | Colombia | MANIZALES | 5.029 | -75.398 | 2610 | 2098 | 9.05 | 7.5 | 1999 | 6.9 | EA |
| 34 | Colombia | Montañitas | 6.625 | -75.66278 | 2970 | 2672 | 7.76 | 7 | 2006 | 3 | AD |
| 35 | Colombia | MONTEVIVO | 6.282 | -75.51 | 2507 | 1786 | 4 | 10.3 | 2002.92 | 6.58 | EA |
| 36 | Colombia | PUERTONARE | 6.127 | -74.672 | 218 | 2426 | 3.54 | 21.7 | 2001.02 | 9.04 | EA |
| 37 | Colombia | RIOBLANCO | 5.044 | -75.442 | 2194 | 1909 | 5.75 | 10.7 | 2002.4 | 2.1 | EA |
| 38 | Colombia | RIOMANSO | 5.561 | -74.702 | 202 | 2152 | 2.97 | 22.2 | 2001.3 | 7.1 | EA |
| 39 | Colombia | Rkalashe | 11.256 | -74.084 | 394 | 1670 | 0.31 | 19.4 | 2007.04 | 4.07 | EA |
| 40 | Colombia | SALENTO | 4.643 | -75.475 | 2540 | 1903 | 6.86 | 8.8 | 1999 | 6.9 | EA |
| 41 | Colombia | SANRAFAEL | 6.287 | -75.051 | 1100 | 3147 | 4.77 | 17.3 | 1999.53 | 10 | EA |
| 42 | Colombia | SANSEBASTIAN | 6.114 | -75.541 | 2696 | 2076 | 5.52 | 9.7 | 2003.16 | 6.95 | EA |
| 43 | Ecuador | Colorado | -3.99025 | -79.965 | 1500 | 1477 | 0.15 | 13.2 | 2005 | 7 | NA, ZA, SB |
| 44 | Ecuador | ElLimo | -3.98436 | -80.1399 | 1380 | 1607 | 0.21 | 14.7 | 2007 | 5 | NA, ZA, SB |
| 45 | Ecuador | GAL10 | -0.84383 | -77.55733 | 1085 | 4452 | 12.24 | 14.2 | 2005.5 | 5.5 | JH |
| 46 | Ecuador | GAL15 | -0.8285 | -77.53917 | 1525 | 4537 | 12.64 | 13.2 | 2005.5 | 5.5 | JH |
| 47 | Ecuador | GUA | -0.63917 | -77.83883 | 1980 | 3143 | 9.94 | 10.8 | 2005.5 | 5.5 | JH |
| 48 | Ecuador | JAS | -1.075 | -77.6075 | 425 | 3677 | 8.63 | 18.3 | 2006 | 5 | JH |
| 49 | Ecuador | OYA35 | -0.1925 | -78.13117 | 3641 | 1384 | 12.61 | 2.3 | 2009.5 | 2.5 | JH |
| 50 | Ecuador | OYA40 | -0.2695 | -78.10833 | 3940 | 1403 | 16.76 | 0.9 | 2009.5 | 2.5 | JH |
| 51 | Ecuador | RHO | -0.67717 | -77.7475 | 1190 | 3998 | 11.82 | 14.2 | 2006 | 5 | JH |
| 52 | Ecuador | SEV | -1.0925 | -77.5095 | 475 | 3619 | 8.26 | 17.9 | 2006 | 5 | JH |
| 53 | Ecuador | SUM15 | -0.6275 | -77.59017 | 1595 | 4226 | 12.49 | 12.4 | 2005.5 | 5.5 | JH |
| 54 | Ecuador | SUM20 | -0.58883 | -77.58867 | 1965 | 3633 | 10.94 | 10.8 | 2005.5 | 5.5 | JH |
| 55 | Peru | PuyuSachaLadera | -11.08634 | -75.42989 | 2100 | 1568 | 2.95 | 9 | 2003 | 3 | RLP |
| 56 | Peru | TrochaUnion1 | -13.11347 | -71.60713 | 3450 | 2448 | 0.63 | 4.33 | 2003 | 4 | KJF, WF |
| 57 | Peru | TrochaUnion2 | -13.11068 | -71.60455 | 3250 | 2677 | 0.63 | 5.742 | 2003 | 4 | KJF, WF |
| 58 | Peru | TrochaUnion3 | -13.10918 | -71.59766 | 3000 | 2884 | 0.69 | 3.3 | 2003 | 4 | KJF, WF |
| 59 | Peru | TrochaUnion4 | -13.10676 | -71.58943 | 2750 | 3228 | 1.29 | 5.4 | 2003 | 4 | KJF, WF |
| 60 | Peru | TrochaUnion5 | -13.09429 | -71.57440 | 2500 | 3457 | 1.96 | 7 | 2003 | 4 | KJF, WF |
| 61 | Peru | TrochaUnion6 | -13.0804 | -71.56587 | 2250 | 3767 | 2.6 | 8.3 | 2003 | 4 | KJF, WF |
| 62 | Peru | TrochaUnion7 | -13.07355 | -71.55890 | 2000 | 4031 | 3.69 | 10.6 | 2003 | 4 | KJF, WF |
| 63 | Peru | TrochaUnion8 | -13.07046 | -71.55569 | 1800 | 4226 | 3.9 | 11 | 2003 | 4 | KJF, WF |
