# Supplementary material for: Large-Scale Patterns of Turnover and Basal Area Change in Andean Forests
Source: PLoS One. 2015 May 14;10(5):e0126594. doi: 10.1371/journal.pone.0126594 (PMC4431807; doi:10.1371/journal.pone.0126594)
Supplement: S2 Table — “Forest group” indicates the classification of the forest plot in one of the groups: HMHFs = Higher Montane Humid Forest, LMMFs = Lower Montane Moist Forests. Country codes: COL = Colomobia, ECU = Ecuador, PER = Peru. Upperscripts on the Plot code indicate the methodological protocol used. The scientific nomenclature was updated according to the Global Biodiversity Information Facility databases (GBIF; www.gbif.org). (DOCX) [file pone.0126594.s002.docx]

**S2 Table. Demographic rates, and dominant or most common species of the 45 permanent plots located in the North-Central Andes.** “Forest group” indicates the classification of the forest plot in one of the groups: HMHFs= Higher Montane Humid Forest, LMMFs= Lower Montane Moist Forests. Country codes: COL= Colomobia, ECU=Ecuador, PER=Peru. Upperscripts on the Plot code indicate the methodological protocol used. The scientific nomenclature was updated according to the Global Biodiversity Information Facility databases (GBIF; www.gbif.org).

| Plot | Country | Plot code | Tree  Turnover  (% yr^-1^) | Tree growth  (m^2^ ha^-1^ yr^-1^) | Relative tree growth  (% yr^-1^) | Basal area  change  (% yr^-1^) | Dominant or most common species in the plot | Family | Forest group |
| --- | --- | --- | --- | --- | --- | --- | --- | --- | --- |
| 1 | COL | Amargal 2 ^a^ | 1.81 | 0.51 | 2.28 | 0.88 | *Welfia regia* H. Wendl. | Arecaceae | HMHFs |
| 2 | COL | Angelopolis ^a^ | 1.20 | 0.64 | 1.45 | - |  |  | - |
| 3 | COL | Besotes ^a^ | 1.90 | 0.32 | 1.75 | 1.39 | *Hura crepitans* L. | Euphorbiaceae | LMMFs |
| 4 | COL | Betulia 1 ^a^ | 1.76 | 0.35 | 2.05 | 0.05 | *Alfaroa colombiana* Lozano & Espinal | Juglandaceae | LMMFs |
| 5 | COL | Betulia 2 ^a^ | 2.07 | 0.36 | 2.29 | -0.21 | *Alfaroa colombiana* Lozano & Espinal | Juglandaceae | LMMFs |
| 6 | COL | Cimitarra ^a^ | 2.75 | - | - | - | *Gustavia longifuniculata* S.A.Mori | Lecythidaceae | - |
| 7 | COL | Combeima ^a^ | 2.76 | - | - | - | *Miconia gleasoniana* Wurdack | Melastomataceae | - |
| 8 | COL | El Ceibal ^a^ | 1.15 | 0.39 | 2.77 | 2.03 | *Bursera simaruba* (L.) Sarg. | Burseraceae | LMMFs |
| 9 | COL | El Ceibal2 ^a^ | 2.39 | - | - | - | *Cordia gerascanthus* L. | Boraginaceae | - |
| 10 | COL | El Diviso ^a^ | 2.10 | 0.33 | 1.99 | -0.11 | *Protium hebetatum* D.C. Daly | Burseraceae | LMMFs |
| 11 | COL | El Rasgon ^a^ | 1.66 | 0.5 | 2.11 | 0.61 | *Beilschmiedia* sp. | Lauraceae | LMMFs |
| 12 | COL | Farallones- E ^a^ | 1.38 | - | - | - | *Alfaroa colombiana*  Lozano & Espinal | Juglandaceae | - |
| 13 | COL | Farallones -U ^a^ | 2.17 | - | - | - | *Cyathea caracasana* Domin | Cyatheaceae | - |
| 14 | COL | La forzosa-A ^a^ | 1.26 | 0.42 | 1.82 | 1.46 |  |  | LMMFs |
| 15 | COL | Manizales ^a^ | 2.17 | - | - | - | *Cordia cylindrostachya* (Ruiz &Pav.) Roem. & Schult. | Boraginaceae | LMMFs |
| 16 | COL | Montañitas ^a^ | 1.76 | 0.45 | 1.48 | 1.78 |  |  | LMMFs |
| 17 | COL | Montevivo ^a^ | 2.60 | 0.37 | 2.86 | 2.86 | *Drimys granadensis* L.f. | Winteraceae | LMMFs |
| 18 | COL | Puerto Nare ^a^ | 3.20 | 0.43 | 4.16 | 1.72 | *Gustavia hexapetala* Sm. | Lecythidaceae | LMMFs |
| 19 | COL | RHO ^a^ | 1.03 | 0.75 | 1.62 | 0.38 |  |  | HMHFs |
| 20 | COL | Rio Blanco ^a^ | 3.13 | - | - | - | *Oreopanax floribundus* (Kunth) Decne. & Planch. | Araliaceae | - |
| 21 | COL | Rio Manso ^a^ | 3.03 | - | - | - | *Astrocaryum* *triandrum* Galeano, R.Bernal & F.Kahn | Arecaceae | - |
| 22 | COL | Rkalashe ^a^ | 2.68 | - | - | - | *Brosimum utile* (Kunth) Pittier | Moraceae | - |
| 23 | COL | Salento ^a^ | 1.31 | - | - | - | *Clidemia* sp. | Melastomataceae | - |
| 24 | COL | San Rafael ^a^ | 3.04 | 0.27 | 2.89 | -0.71 | *Miconia elata* DC. | Melastomataceae | LMMFs |
| 25 | COL | San Sebastian ^a^ | 1.63 | 0.23 | 1.62 | -0.35 | *Clethra fagifolia* Kunth | Clethraceae | LMMFs |
| 26 | ECU | El Limo^a^ | 1.65 | 0.52 | 3.48 | - | *Ficus cervantesiana* Standl. & L.O.Williams | Moraceae | - |
| 27 | ECU | Colorado^a^ | 2.93 | 0.48 | 2.36 | 0.95 | *Otoba parvifolia* (Mgf.) A.H.Gentry | Lauraceae | LMMFs |
| 28 | ECU | GAL10^a^ | 2.20 | 0.62 | 1.39 | 0.28 | *Iriartea deltoidea* Ruiz & Pav. | Arecaceae | HMHFs |
| 29 | ECU | GAL15^a^ | 0.91 | 0.32 | 0.95 | -0.59 | *Dictyocaryum lamarckianum* (Mart.) H. Wendl. | Arecaceae | HMHFs |
| 30 | ECU | GUA^a^ | 2.84 | 0.38 | 1.70 | -1.72 | *Weinmannia pinnata* L. | Cunoniaceae | HMHFs |
| 31 | ECU | JAS^a^ | 1.06 | 0.5 | 1.50 | 0.94 | *Otoba parvifolia* (Markgr.) A.H. Gentry | Myristicaceae | LMMFs |
| 32 | ECU | OYA35^a^ | 0.41 | - | - | - | *Polylepis pauta* Hieron. | Rosaceae |  |
| 33 | ECU | OYA40^a^ | 0.25 | 0.12 | 0.45 | 0.43 | *Polylepis pauta* Hieron. | Rosaceae | HMHFs |
| 34 | ECU | SEV^a^ | 1.26 | 0.56 | 1.75 | 0.25 | *Pseudosenefeldera inclinata* (Müll. Arg.) Esser | Euphorbiaceae | LMMFs |
| 35 | ECU | SUM15^a^ | 1.63 | 0.35 | 0.70 | -1.25 | *Clarisia biflora* Ruiz & Pav. | Moraceae | HMHFs |
| 36 | ECU | SUM20^a^ | 1.16 | 0.43 | 1.73 | -2.44 | *Chrysochlamys membranacea* Planch. & Triana | Clusiaceae | HMHFs |
| 37 | PER | Puyu Sacha Ladera^a^ | 2.08 | 0.92 | 3.87 | - | *Miconia aureoides* Cogn. | Melastomataceae | - |
| 38 | PER | Trocha Union 1^a^ | 1.35 | 0.25 | 0.92 | 0.29 | *Weinmannia cochensis* Hieron. | Cunoniaceae | HMHFs |
| 39 | PER | Trocha Union 2^a^ | 1.47 | 0.31 | 1.12 | 0.73 | *Weinmannia bangii* (Rusby) Engl. | Cunoniaceae | HMHFs |
| 40 | PER | Trocha Union 3^a^ | 1.39 | 0.21 | 0.97 | 1.11 | *Clusia alata* Planch. & Triana | Clusiaceae | HMHFs |
| 41 | PER | Trocha Union 4^a^ | 1.66 | 0.37 | 1.50 | 0.02 | *Clusia sphaerocarpa* Planch. & Triana | Clusiaceae | HMHFs |
| 42 | PER | Trocha Union 5^a^ | 1.70 | 0.3 | 1.23 | -0.12 | *Alchornea grandiflora* Müll. Arg. | Euphorbiaceae | HMHFs |
| 43 | PER | Trocha Union 6^a^ | 2.40 | 0.36 | 1.70 | -0.71 | *Alzatea verticillata* Ruiz & Pav. | Alzateaceae | HMHFs |
| 44 | PER | Trocha Union 7^a^ | 1.55 | 0.25 | 1.48 | 0.69 | *Alzatea verticillata* Ruiz & Pav. | Alzateaceae | HMHFs |
| 45 | PER | Trocha Union 8^a^ | 2.54 | 0.31 | 1.37 | -1.05 | *Clethra revoluta* Ruiz & Pav. | Clethraceae | HMHFs |

^a^ Protocol used: Phillips, O. & Baker, T. 2002, Manual de Campo para la Remedición y Establecimiento de Parcelas. RAINFOR. Sixth Framework Programme (2002-2006).<http://www.eci.ox.ac.uk/research/ecodynamics/panamazonia/spanish/rainfor_field_manual_spanish.pdf>
