# Supplementary material for: Large-Scale Patterns of Turnover and Basal Area Change in Andean Forests
Source: PLoS One. 2015 May 14;10(5):e0126594. doi: 10.1371/journal.pone.0126594 (PMC4431807; doi:10.1371/journal.pone.0126594)
Supplement: S3 Table — Scientific nomenclature after the Global Biodiversity Information Facility databases (GBIF; www.gbif.org). (DOCX) [file pone.0126594.s003.docx]

**S3 Table. Demographic rates, and dominant or most common species of the 18 permanent plots located in South-Western Argentina.** Scientific nomenclature after the Global Biodiversity Information Facility databases (GBIF; www.gbif.org).

| Plot | Plote code | Turnover  (%) | Growth  (m^2^ ha^-1^ yr^-1^) | Growth  (% yr^-1^) | Basal area  change  (% yr^-1^) | Dominant or most common species | Family | Protocol  used |
| --- | --- | --- | --- | --- | --- | --- | --- | --- |
| 1 | Mi1 | 3.17 | 0.71 | 0.52 | 0.56 | *Solanum aligerum* Schltdl. | Solanaceae | Malizia et al. 2003 |
| 2 | Mi2 | 1.62 | 0.56 | 0.14 | -0.24 | *Myrcianthes mato* (Griseb.) McVaugh | Myrtaceae | Malizia et al. 2003 |
| 3 | Ha1 | 1.80 | 0.36 | 0.27 | 0.55 | *Eugenia uniflora* L. | Myrtaceae | Malizia et al. 2003 |
| 4 | Ha2 | 1.84 | 0.36 | 0.24 | -0.08 | *Eugenia uniflora* L. | Myrtaceae | Malizia et al. 2003 |
| 5 | Ha3 | 1.46 | 0.39 | 0.28 | -0.3 | *Eugenia uniflora* L. | Myrtaceae | Malizia et al. 2003 |
| 6 | Ha4 | 2.29 | 0.28 | 0.26 | 0.46 | *Piper tucumanum* C.DC. | Piperaceae | Malizia et al. 2003 |
| 7 | Ha5 | 3.57 | 0.68 | 0.41 | 1.04 | *Eugenia uniflora* L. | Myrtaceae | Malizia et al. 2003 |
| 8 | Ha6 | 3.20 | 0.44 | 0.74 | -0.23 | *Piper tucumanum* C.DC. | Piperaceae | Malizia et al. 2003 |
| 9 | Sur | 2.46 | 0.36 | 0.32 | 0.74 | *Piper tucumanum* C.DC. | Piperaceae | Malizia et al. 2003 |
| 10 | Norte | 2.68 | 0.27 | 0.6 | 0.6 | *Terminalia triflora* Lillo | Combretaceae | Malizia et al. 2003 |
| 11 | Tabacal500 | 2.05 | 0.4 | 0.32 | 0.68 | *Trichilia clausseni* C.DC. | Meliaceae | Condit 1998 |
| 12 | Tecpetrol500 | 2.31 | 0.43 | 0.21 | 1.03 | *Phyllostylon rhamnoides* (J.Poiss.) Taub. | Ulmaceae | Condit 1998 |
| 13 | Yuchan500 | 1.18 | 0.38 | 0.18 | -1.09 | *Phyllostylon rhamnoides* (J.Poiss.) Taub. | Ulmaceae | Condit 1998 |
| 14 | AbraGrande550 | 5.54 | 0.43 | 0.18 | 3.48 | *Parapiptadenia excelsa* (Griseb.) Burkart | Fabaceae | Condit 1998 |
| 15 | SanMartin600 | 1.46 | 0.32 | 0.23 | -0.28 | *Anadenanthera colubrina* (Vell.) Brenan | Fabaceae | Condit 1998 |
| 16 | ValleMorado650 | 2.43 | 0.37 | 0.39 | 0.38 | *Trichilia clausseni* C.DC. | Meliaceae | Condit 1998 |
| 17 | RíoSecoII | 2.33 | 0.31 | 0.42 | 1.17 | *Anadenanthera colubrina* (Vell.) Brenan | Fabaceae | Condit 1998 |
| 18 | Km25 | 2.18 | 0.28 | 0.21 | 1.38 | *Diatenopteryx sorbifolia* Radlk. | Sapindaceae | Condit 1998 |

Malizia, L., T. Easdale, A. Malizia, H. R. Grau, J. Carilla, A. Brown y T. Lomascolo. 2003. No publicado. RedSPP : Red Subtropical de Monitoreo Forestal. Laboratorio de Investigaciones Ecologicas de las Yungas, Universidad Nacional de Tucumán y Fundación ProYungas. 30 pp.

Condit, R. 1998. Field guide for tropical forest census plots: methods and results from Barro Colorado Island, Panama and a comparison with other plots. R. G. Landes Co. 211 pp.
