# Supplementary material for: Large-Scale Patterns of Turnover and Basal Area Change in Andean Forests
Source: PLoS One. 2015 May 14;10(5):e0126594. doi: 10.1371/journal.pone.0126594 (PMC4431807; doi:10.1371/journal.pone.0126594)
Supplement: S4 Table — (DOCX) [file pone.0126594.s004.docx]

**S4 Table. Results of the Linear regression analyses using PCA factors as predictors of forest demography in North-Central Andean, and North-Western Argentina permanent forest plots.**

|  | Northern Andes | |  |  |  |  |  |  |  |
| --- | --- | --- | --- | --- | --- | --- | --- | --- | --- |
|  | **Term** | **Estimate** | **Std Error** | **t Ratio** | ***P*** | **DF** | ***F*** | ***P*** | ***R^2^*** |
| Tree turnover (% yr^-1^) | Intercept | 1.91 | 0.15 | 13.11 | <0.001 | 45 | 0.14 | 0.712 | 0.003 |
|  | PCA 1 | -0.04 | 0.12 | -0.37 | 0.712 |  |  |  |  |
| Tree growth (m^2^ yr^-1^) | Intercept | 0.38 | 0.03 | 11.54 | <0.001 | 33 | 1.34 | 0.254 | 0.04 |
|  | PCA 1 | 0.02 | 0.02 | 1.16 | 0.254 |  |  |  |  |
| Relative tree growth (% yr^-1^) | Intercept | 1.89 | 0.18 | 10.03 | <0.001 | 33 | 0.42 | 0.517 | 0.01 |
|  | PCA 1 | -0.09 | 0.14 | -0.65 | 0.517 |  |  |  |  |
| Basal area net change (% yr^-1^) | Intercept | 0.84 | 0.29 | 2.9 | 0.006 | 32 | 3.41 | 0.074 | 0.09 |
|  | PCA 1 | -0.39 | 0.21 | -1.85 | 0.074 |  |  |  |  |
| Tree turnover (% yr^-1^) | Intercept | 1.9 | 0.1 | 19.41 | <0.001 | 45 | 9.77 | 0.003 | 0.19 |
|  | PCA 2 | -0.21 | 0.07 | -3.12 | 0.003 |  |  |  |  |
| Tree growth (m^2^ yr^-1^) | Intercept | 0.41 | 0.02 | 15.75 | <0.001 | 33 | 4.24 | 0.047 | 0.11 |
|  | PCA 2 | -0.04 | 0.02 | -2.06 | 0.047 |  |  |  |  |
| Relative tree growth (% yr^-1^) | Intercept | 1.92 | 0.12 | 14.8 | <0.001 | 33 | 15.26 | <0.001 | 0.32 |
|  | PCA 2 | -0.38 | 0.09 | -3.91 | <0.001 |  |  |  |  |
| Basal area net change (% yr^-1^) | Intercept | 0.61 | 0.24 | 2.53 | 0.016 | 32 | 2.93 | 0.096 | 0.08 |
|  | PCA 2 | -0.31 | 0.18 | -1.71 | 0.096 |  |  |  |  |

|  | Austral Andes | |  |  |  |  |  |  |  |
| --- | --- | --- | --- | --- | --- | --- | --- | --- | --- |
|  | **Term** | **Estimate** | **Std Error** | **t Ratio** | ***P*** | **DF** | ***F*** | ***P*** | ***R^2^*** |
| Tree turnover (% yr^-1^) | Intercept | 4.68 | 4.21 | 1.11 | 0.283 | 18 | 0.29 | 0.599 | 0.02 |
|  | PCA 1 | 1.13 | 2.11 | 0.54 | 0.599 |  |  |  |  |
| Tree growth (m^2^ yr^-1^) | Intercept | -0.35 | 0.5 | -0.71 | 0.489 | 18 | 2.33 | 0.146 | 0.12 |
|  | PCA 1 | -0.38 | 0.25 | -1.53 | 0.146 |  |  |  |  |
| Relative tree growth (% yr^-1^) | Intercept | 1.92 | 1.90 | 1.01 | 0.328 | 18 | 0.02 | 0.879 | 0.001 |
|  | PCA 1 | 0.15 | 0.95 | 0.15 | 0.879 |  |  |  |  |
| Basal area change (% yr^-1^) | Intercept | -8.50 | 3.49 | -2.43 | 0.027 | 18 | 5.66 | 0.030 | 0.26 |
|  | PCA 1 | -4.16 | 1.75 | -2.38 | 0.030 |  |  |  |  |
| Tree turnover (% yr^-1^) | Intercept | 2.33 | 0.33 | 7.13 | 0.000 | 18 | 0.17 | 0.686 | 0.01 |
|  | PCA 2 | -0.26 | 0.64 | -0.41 | 0.686 |  |  |  |  |
| Tree growth (m^2^ yr^-1^) | Intercept | 0.46 | 0.03 | 13.32 | 0.000 | 18 | 6.33 | 0.023 | 0.28 |
|  | PCA 2 | 0.17 | 0.07 | 2.52 | 0.023 |  |  |  |  |
| Relative tree growth (% yr^-1^ | Intercept | 1.72 | 0.14 | 11.94 | 0.000 | 18 | 0.82 | 0.377 | 0.05 |
|  | PCA 2 | 0.25 | 0.28 | 0.91 | 0.377 |  |  |  |  |
| Basal area change (% yr^-1^) | Intercept | 0.11 | 0.29 | 0.37 | 0.714 | 18 | 2.48 | 0.135 | 0.13 |
|  | PCA 2 | 0.90 | 0.57 | 1.57 | 0.135 |  |  |  |  |
