# Supplementary material for: Large-Scale Patterns of Turnover and Basal Area Change in Andean Forests
Source: PLoS One. 2015 May 14;10(5):e0126594. doi: 10.1371/journal.pone.0126594 (PMC4431807; doi:10.1371/journal.pone.0126594)
Supplement: S1 File — (DOCX) [file pone.0126594.s005.docx]

**S1 File. Ethics statement.**

Argentina: Plots 1-10. No specific research permit was necessary. These plots were located within the Parque Biológico Sierra de San Javier, which belongs to the Universidad Nacional de Tucumán. Contact person: Agustina Malizia. No endangered or protected species were present in the study plots. Plots 11-18. Secretaría de Ambiente de Salta, Secretaría de Gestión Ambiental de Jujuy y Administración de Parques Nacionales provided research permits to collect data in Salta and Jujuy. The plots are located in private properties. Contact person: Cecilia Blundo and Lucio Malizia. Some of the plots included the protected tree *Amburana cearensis*. Colombia: Plots 19-21. No specific research permit was necessary. Contact organization: Corporación Regional del Centro y Norte de Antioquia (CORANTIOQUIA). No endangered or protected species were present in the study plots, as far as we know. Plots 22-42. No specific research permit was necessary. Plots were established in private lands belonging to various different persons. Contact person: Esteban Álvarez. No endangered or protected species were present in the study plots, as far as we know. Ecuador: Plots 43-44: Research permits were issued by the Ministerio de Ambiente, Loja, signed by Carlos Espinosa. Both plots are located within private properties. Contact person: Zhofre Aguirre. No endangered or protected species were present in the study plots, as far as we know. Plots 45-54. All permits were issued by the Dirección Nacional de Biodiversidad of the Ministerio de Ambiente del Ecuador. Plots 45, 46, 51, 53, 54: Parque Nacional Sumaco-Galeras and Sumaco biosphere reserve. Plots 49, 50: Reserva Ecológica Cayambe-Coca. Plots 48, 52: private reserves within Sumaco biosphere reserve. Plots 47 Reserva Ecológica Antisana and Sumaco biosphere reserve. Contact person: Juergen Homeier. Plots contained endengared species. Peru: Plot 55. No specific research permit was necessary. The plot was located in private property. Contact person: Reynaldo Linares-Palomino. No endangered or protected species were present in the study plots, as far as we know. Plots 56-63 Permits were issued by the Instituto Nacional de Recursos Naturales (IRENA) and Servicio Nacional de Áreas Naturales Protegidas por el Estado, Ministerio del Ambiente del Perú (SERNANP). These plots were located within the Parque Nacional Manu. No endangered or protected species were present in the study plots, as far as we know. For details on plot numbers refer to Supplementary information 1.
