# Supplementary figures and images for: Large-Scale Patterns of Turnover and Basal Area Change in Andean Forests
Source: PLoS One. 2015 May 14;10(5):e0126594. doi: 10.1371/journal.pone.0126594 (PMC4431807; doi:10.1371/journal.pone.0126594)

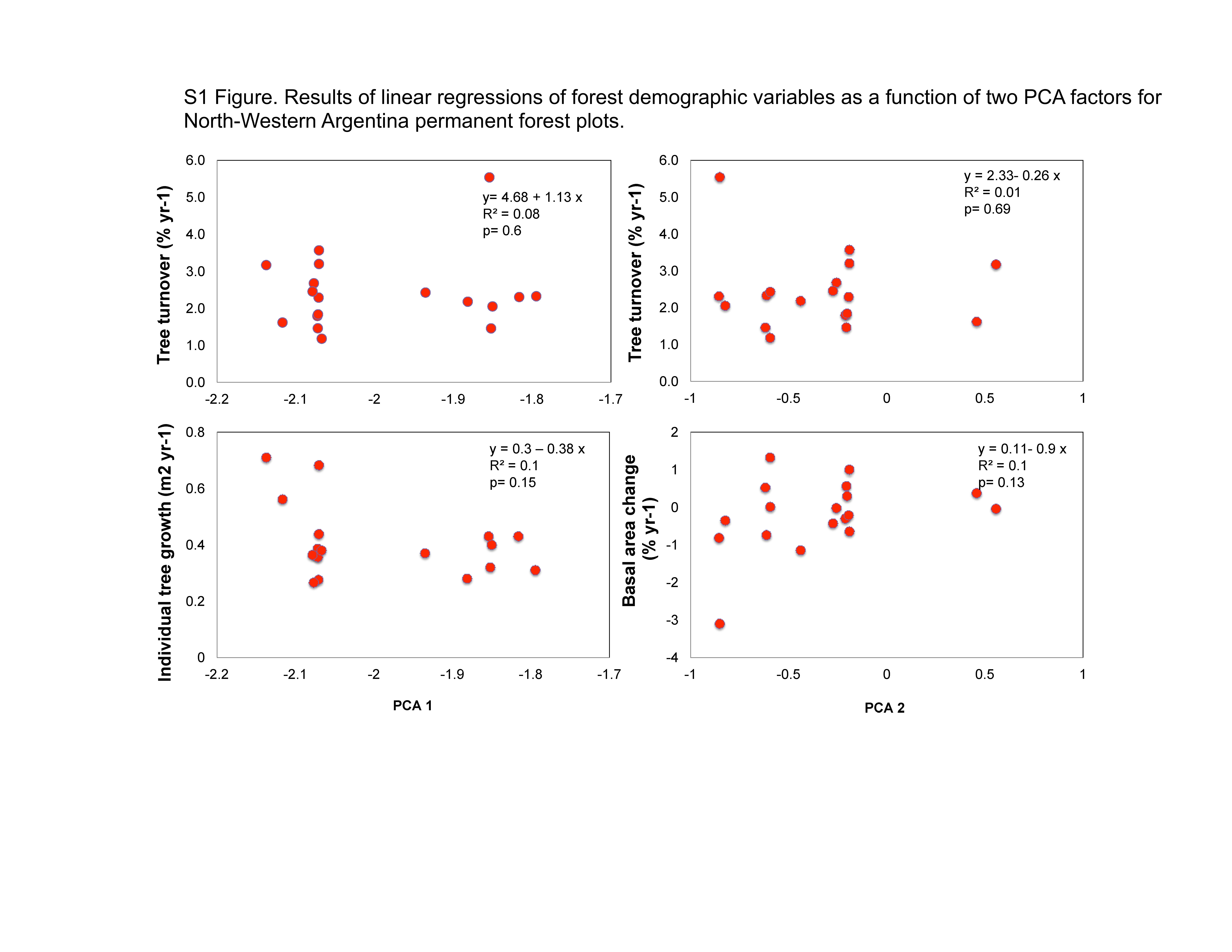

Supplement: S1 Fig — (TIFF) [file pone.0126594.s006.tiff]
